# Ethnobotanical remarks on Central and Southern Italy

**DOI:** 10.1186/1746-4269-3-23

**Published:** 2007-05-30

**Authors:** Paolo Maria Guarrera, Leporatti Maria Lucia

**Affiliations:** 1 Museo Nazionale Arti e Tradizioni Popolari, Roma, Italy; 2 Dipartimento di Biologia Vegetale Università "La Sapienza" Roma, Italy

## Abstract

**Background:**

The present paper is a brief survey on the ethnobotanical works published by the Authors since 1981, concerning the research carried out in some southern and central Italian regions. Before Roman domination these territories were first inhabited by local people, while the southern areas were colonized by the Greeks. These different cultural contributions left certain traces, both in the toponyms and in the vernacular names of the plants and, more generally, in the culture as a whole.

**Methods:**

Field data were collected through open interviews, mainly of farmers, shepherds and elderly people, born or living in these areas for a long time. Voucher specimens of collected plants are preserved in the respective herbaria of the Authors and in the herbarium of "Roma Tre" University. Important contributions have been made by several students native to the areas under consideration. A comparative analysis with local specific ethnobotanical literature was carried out.

**Results:**

The paper reports several examples concerning human and veterinary popular medicine and in addition some anti-parasitic, nutraceutic, dye and miscellaneous uses are also described. Moreover vernacular names and toponyms are cited. Eight regions of central and southern Italy (particularly Latium, Abruzzo, Marche and Basilicata) were investigated and the data obtained are presented in 32 papers. Most of the species of ethnobotanical interest have been listed in Latium (368 species), Marche (274) and Abruzzo (203). The paper also highlights particularly interesting aspects or uses not previously described in the specific ethnobotanical literature.

**Conclusion:**

Phyto-therapy in central and southern Italy is nowadays practised by a few elderly people who resort to medicinal plants only for mild complaints (on the contrary food uses are still commonly practised). Nowadays therapeutic uses, unlike in the past, are less closely or not at all linked to ritual aspects. Several plants deserve to be taken into consideration not only from the anthropological or cultural point of view, but also for further phyto-chemical investigation. Our studies, as well as those of other authors, try to provide an original picture of the local ethno-biodiversity.

## Background

The sudden evolution of society towards technological patterns and the increasing use of synthetic remedies, has seen a consequent erosion of a rich cultural heritage regarding popular phyto-therapy, that had developed over the centuries. In fact, from the end of XIX century onwards, people engaged in agricultural activities started to leave the countryside and an irreversible process of urbanization began. In the same period the first works on popular medicine were published by anthropologists who were also doctors ([1-8] etc.) These researchers highlighted the superstitious and ritual aspects of folk medicine but, on the other hand, reported several recipes involving medicinal plants, some of which (such as some vernacular names) still remain valid today (e.g. [3,9]). Nevertheless these first works are sometimes lacking in details regarding the manipulation or the drugs involved, and sometimes even the scientific name of the species is incorrect. The aim of these first works was to highlight local customs as national values. At the beginning of the XX century the ethnologist Lamberto Loria (1855–1913), founder of the National Museum of Arts and Folk Traditions in Rome, already realized that the progressive abandoning of the rural way of life could have a marked negative impact on folk traditions. So, he started to collect numerous documents of material culture such as folk costumes, pottery and many artefacts made from plants such as baskets, amulets, work tools, shepherd's crooks etc. In the National Museum of Arts and Folk Traditions in Rome many artefacts made with plant materials are preserved, but it is not always possible to trace back to the botanical species from which they were made. Loria intended the collection of artefacts to serve as a tool for gaining knowledge of people's lives and traditions. On the contrary, Pitré [10] (professor of Demopsychology at Palermo University) considered it more important to gather stories, fairy tales, superstitions and recipes, that is to say those oral traditions that are today defined as "immaterial goods". Today, modern research holds these two lines of folk traditions to be equally important in order to be able to document the way of life of a people: gathering documents of "material culture" (or portraying these with photos, drawings etc.) and collecting information on the oral traditions. Unfortunately, in the papers of these studies, many plants are often cited only with their vernacular names and they consequently cannot be identified from a scientific, botanical point of view. It is, however, at least possible to attempt to determine the botanical genus from the Italian or vernacular name, but not the species. In the last thirty years, after a long period of oblivion, there has been a reawakening of these studies from the anthropological point of view [11].

In the second half of XX century, the first modern ethnobotanical studies were carried out in Italy ([12-16] etc.), where the species involved in the folk traditions were precisely determined by their botanical names. Following on the trail of these work, from the late nineteen seventies on, we carried out ethnobotanical studies in different central and southern Italian regions. The studies were mainly focused on medicinal folk uses, ethno-veterinary practices, food uses, handicraft and home-made uses; ritual-magic uses were considered to a marginal extent. These research projects were made in order to highlight the elements of the local ethno-biodiversity and cultural heritage of the past, quickly disappearing in our changing society. The scientific interest of these uses and the relative potential economic applications go well with man's need to maintain a sense of his own identity related to his historical and geographical environment.

In central and southern Italy, the imposing Apennine mountains alternate with flat coastal belts such as the Maremma, the Cilento plain, Salento etc. These areas are surrounded by three sections of the Mediterranean sea: the Tyrrhenian, Ionian and Adriatic seas.

In the coastal areas the climate is typically Mediterranean with dry summers, rainy autumns and springs and temperate winters, whilst in the inland mountainous areas the winter is harsher and longer, and the summer hot. Before Roman domination these territories were first inhabited by local people, while the southern areas were colonized by the Greeks. These different cultural contributions left certain traces, both in the toponyms and in the vernacular names of the plants and, more generally, in the culture as a whole, traces still visible today. Since ancient times, people have been engaged in agricultural activities, in spite of the fact that in some areas the landscape is prevailingly mountainous.

## Methods

The present paper presents a survey of our studies published from 1981 up to the present day (some of them are still in progress). Our research started in the Vegetal Biology Department of the University "La Sapienza" of Rome.

A series of interviews was conducted, mainly with farmers, shepherds and elderly people, born or living in these areas for a long time. According to the different cases, open, structured or semi-structured interviews were carried out with the purpose of collecting the greatest possible number of data in the various fields since the information was in the process of disappearing. The informants are aware that the information they have provided will be published. Voucher specimens of exsiccata were collected and preserved in the respective herbaria of the Authors, or in the herbarium of Roma Tre" University. Important contributions were made by several students native to the areas in question. The collected data were necessarily supported by a comparative analysis with local specific ethnobotanical literature, when present; and the information about drugs, therapeutic uses and manipulations have been reported for every species recorded, such as the vernacular names. In recording information, care was taken not to "translate" this into medical terms, but to refer exactly whatever was reported, so as to avoid the information being in any way falsified. In the latest studies the number of informants for each use has been also annotated "quantitative ethno-botany". Nevertheless, the authors would also like to stress that data collected with the "ethnographic method" can be significant, if the same uses are reported in different villages and/or regions. The fidelity level (FL) was calculated in only one work, written from precisely this perspective [17], on the major use of plants in Latium, Abruzzo and Marche, as proposed by Friedman et al. [18], considering not the number of informants but of localities, (FL = the ratio between number of localities reported for the primary uses of one species and number of localities where the use of this species is reported).

## Results

Our research can be summarised in Table 1.

**Table 1:** Research in central-southern Italy by Guarrera and Leporatti

**Investigated region**	**Published papers**	**Papers at press or in progress**	**Number of species **^(^*^)^
Abruzzo	6		203
Apulia	1	1	98
Basilicata	6		124
Calabria	3		190
Latium	9		368
Marche	3		274
Molise	1	1	17
Umbria	1		33

(*) The number of species is calculated only on the basis of the published works

Twenty-nine miscellaneous papers concerning various aspects of the folk plant traditions have been published, in addition to those in the table 1.

Our studies (often in collaboration with other authors) covered almost all of the Latium, Marche and Abruzzo regions; on the contrary Calabria, Basilicata and Apulia have still been scarcely examined and further studies are still in progress (table 2). The criterion of reporting the number of informants was adopted only in the most recent works, so this datum is not available for each use reported in the present paper.

**Table 2:** Research in central-southern Italy by other authors (before 1950: notable ethnoanthropological papers; after 1950: ethnobotanical papers) carried out.

	**Abruzzo**	**Apulia**	**Basilicata**	**Calabria**	**Latium**	**Marche**	**Molise**	**Umbria**
Papers **before 1950**	3	-	-	-	1	3	-	1
Papers **after 1950**	3	3	6	1	1	3	-	2

### A) Human therapy

Phytotherapy in central and southern Italy is nowadays practised by only a few elderly people, who resort to medicinal plants only for minor ailments; e.g. no cardiotonic plants are used and toxic plants have only an external use.

Some medicinal plants are cited not previously quoted in Gastaldo [19] (the only summary of Italian ethnopharmacobotanical literature until 2006, when the compendium by Guarrera was published [20]).

Owing to the complexity of the picture that emerges from these studies, only few examples of new and peculiar remedies, or rare uses can be given for each investigated region.

The more interesting species are then discussed in relation to their active compounds and phyto-therapeutic properties.

#### Abruzzo

***Cirsium arvense ***(L.) Scop. [Asteraceae]. The juice of the leaves, locally applied, heals wounds. The same use is present also in Marche region [16,21-23]. The vulnerary use had not previously been cited in the principal pharmacobotany texts. Taraxasterol, aplotaxene and their derivatives, in addition to flavonoids are present in the plant [24-27]; an UV-mediated antibiotic activity was reported for this species [28].

***Fraxinus ornus ***L. [Oleaceae]. A decoction of leaves is drunk in cases of arthritis and gout [9]. In Abruzzo [16] an analogous use (hot poultices of a decoction of leaves) is reported. Coumarins are present in the leaves, mainly esculetol-7-glycosyde [29].

#### Apulia

***Phlomis fruticosa ***L. [Lamiaceae]. A Mediterranean species widespread only in Apulia, Calabria, Sicily and Sardinia; it is esteemed as an anti-tussive [30]. Active substances and therapeutical action have not been found in the consulted literature.

***Marrubium vulgare ***L. [Lamiaceae]. It was used in case of rheumatism, haemorrhoids, wounds and even as an anti-malarial [30], as reported also by Bianchi and Gallifuoco [31] together with other uses. Marrubin, essential oil, saponins and bitter substances are present. This species is considered as a "panacea" in southern Italy, and it would require further studies on the great quantities of uses attributed in folk traditions also in other Italian areas.

#### Basilicata

***Acer neapolitanum ***Ten. [Aceraceae]. An endemic species of southern Italy, its bark is used in first aid as a bandage in case of wounds [32]. According to the updated nomenclature by Conti et al. [33]*A. neapolitanum *is included in *Acer opalus *Mill. subsp. *obtusatum *(Waldst. & Kit. ex Willd.) Gams. The tannin content helps the action of the bark. The use of fresh bark as a bandage is exclusive to the area of Maratea (Basilicata) and had never been described before in central Italy for the same purpose ***Ulmus minor ***Mill. [Ulmaceae] bark had been commonly applied [29], and in the northern-eastern Alps *Larix decidua *bark instead [34].

#### Calabria

***Genista anglica ***L. [Fabaceae], widespread in the heart of Central Europe, present in Italy only in this region, it had been employed as a diuretic and for kidney stones; a decoction of the aerial part cured ulcers (it has note been determined whether we are dealing with gastric or duodenal ulcers) [35]. This plant contains flavonoids and alkaloids [36], but it is not clear which chemical substances may be responsible for the reported effects.

***Laurus nobilis ***L. [Lauraceae]. In the past, an infusion of leaves was widely used by women who had just given birth as a galactagogue (5 informants) [37].

***Pinus laricio ***Poiret [Pinaceae]. This species is endemic in Calabria and in Sicily; it is employed in mixture with other plants for stomach ache [35]. The essential oil it contains (terpenes) justifies this use.

***Quercus frainetto ***Ten. [Fagaceae], in a mixture with other species it is an anti-rheumatic and lenitive for burns [35]. Mainly tannins are present.

***Scrophularia canina ***L. [Scrophulariaceae] is described in the area of Crotone for several external uses (5 informants): to treat rhagades on the breast, wounds and haematomas, to bring out abscesses and fistulas (generally the plant was boiled and the decoction employed as an antiseptic wash) [37]. The plant contains iridoid glycosides [38].

#### Latium

***Centaurea bracteata ***Scop. [Asteraceae]. Mixed with *C. calcitrapa *is anti-tussive and a diuretic [29]. The chemical compounds are similar to those of *Centaurea pratensis*.

***Centaurium erythraea ***Rafn [Gentianaceae]. It is a hypotensive [21] and antimalarial, this last use being recorded also for Apulia [31]. It contains erytro-centaurin, gentiopicrin, fitosterin etc., but it is not known precisely which active principle is responsible for the hypotensive action.

***Lupinus luteus ***L. [Fabaceae]. A rare species in central-southern Italy and islands, it is known as a hypotensive [29]. Its active principles (lupinin, lupanin, lupinic acid) are similar to those of the better known *Lupinus albus*.

***Ranunculus ficaria ***L. [Ranunculaceae], in Latium (Acquapendente) the small tubers are browned in oil and then applied locally to cure haemorrhoids (4 informants)[39]. This use is known for other countries and also in Sicily, where the vernacular name is "pianta pi emorroidi" [40-42].

***Silene italica ***(L.) Pers. [Caryophyllaceae]. Fumigations with seeds on embers to cure sore throat [29]. Ecdysteroids were isolated in this species, with adaptogenic effects [43-45]

Several new uses of wild or cultivated species are cited by Guarrera [29] and their active principles and properties are named and discussed.

#### Marche

***Centaurea pratensis ***Thuil. [Asteraceae]. It is a cholagogue and stomachic. The content in centaurin and bitter substances can justify the activity [46].

***Chelidonium majus ***L. [Papaveraceae]. The latex was used as a cicatrising agent for sores resistant to usual medicaments [21]. This plant contains spartein, chelidoxantin, protopin, berberin and other alkaloids, but the active principle responsible for this action has to be further investigated.

***Euphorbia paralias ***L. [Euphorbiaceae]. The latex was used as an anaesthetic by fishermen stung by weever fish (*Trachynus vipera*) [47]. Anaesthetic activity was demonstrated for an exotic *Euphorbia *species [48].

***Marrubium incanum ***Desr. [Lamiaceae], it is used as an emmenagogue; it is also considered useful in losing weight [46] and considered effective in liver colic [21]. Their active substances are similar to those of *M. vulgare*.

#### Interesting uses

***Sambucus nigra ***L. [Caprifoliaceae] pith was used in ointments for burns boiled with olive oil and beeswax in Latium and Abruzzo [9,29] (see also [16]). The active principles of the pith (triterpenes such as ursolic acid, β-amirin, betulin) can explain the anti-inflammatory effects and the vulnerary action of this preparation. This folk use is reported by a discreet number of informants.

In Abruzzo and Latium. For removing thorns from hands, hot scales of ***Allium cepa ***L. [Liliaceae] ([29], see also [16]) and ***Ocimum basilicum ***L. [Lamiaceae ] leaves [29] are applied mixed with olive oil. The manipulation is lenitive for the skin.

***Myrtus communis ***L. [Myrtaceae], one of the most characteristic Mediterranean species. In Basilicata and Calabria, the burnt and powdered leaves were applied to reddened skin in children and also used to prevent reddening of the feet [32,49]. The use is widespread in other southern Italian and Mediterranean areas, e.g. Campania, Sicily, Sardinia [42,50,51]. It contains myrtenol, terpenes, tannins and resins.

Fruits of ***Tamus communis ***L. [Discoreaceae], in central Italy are rubbed onto the skin for rheumatic and arthritic pains [29] (see also [23] etc.). Diosgenin, β-sitosterol, stigmasterol, campesterol found in *Tamus communis *are responsible for the anti-inflammatory activity.

Some species are considered like a "panacea":

***- Malva sylvestris ***L. [Malvaceae] used as a lenitive in a wide range of inflammations, such as toothache, etc.

***- Ruta graveolens ***L. [Rutaceae], in southern Italy; the activity of protecting capillary vessels has been demonstrated. The internal use of *Ruta graveolens *is generally contra-indicated, while there are many, frequent external uses. Its widespread use in the past seems to be linked also to ritual aspects [[22,37] etc.].

In general the therapeutic effects of the plants can be enhanced or reduced according to the combination of various species in mixtures or recipes.

It is of particular note that many plants have several principal folk uses. In a study carried out concerning central Italy [17] several species can be cited e.g.:

*-****Urtica dioica ***L. [Urticaceae], the primary use of the leaves is for gastrointestinal disorders (11 localities), the secondary use is for rheumatic pains (10 loc.).

-***Parietaria diffusa ***Mert. et Koch [Urticaceae], the primary use of the aerial parts is in the case of bruises and sprains (18 loc.), the secondary use in urinary diseases (17 loc.).

-***Allium cepa ***L. [Liliaceae], the primary use of the bulb is for urinary diseases (12 loc.), the secondary for coughs (8 loc.).

Among the more interesting uses present in the Tyrrhenian area of Basilicata [32], we must recall:

*-****Ceratonia siliqua ***L. [Fabaceae] dried seeds in decoction with other plants, for coughs and colds (10 informants).

*-****Leopoldia comosa ***(L.) Parl. [Liliaceae], the grated bulb is applied on a paper disc for compresses in case of toothache (9 inf.), or as above, for headache (5 inf.).

*- ****Plantago major ***L. [Plantaginaceae], crushed leaves or compresses applied to boils (6 inf.).

### B) Veterinary science

On the basis of a recently published ethno-veterinary review [52] the first national data bank of folk veterinary medicine in Europe has been created. Some of these uses are at the present time obsolete. The complaints most commonly treated relate to the digestive system and cutaneous complaints. Many other plants were used to heal wounds and to soothe inflammations caused by harnesses or yokes, etc., while several plant uses are associated with delivery and birth. Sometimes the therapeutic uses are similar to those found in human medicine.

Otherwise particular plants were used:

***Artemisia absinthium ***L. [Asteraceae]. The aerial parts were fodder for young calves to help digest milk in Marche region [21].

***Helleborus bocconei ***Ten. [Ranunculaceae]. In the past, in Calabria, it was the only remedy known by cowherds for treating bronchitis in cattle. The long petiole of the basal leaves was divided into 3 parts and it was inserted in a hole practised on the back of the ear of the animal or under the fur of the lateral part of the neck. In other Italian regions [20] similar methods are described with species of the genus *Helleborus *[37].

***Rubia peregrina ***L. [Rubiaceae] was given, in Latium, as food to assist expulsion of the placenta due to antraquinone compounds [29]. ***Juniperus sabina ***L. [Cupressaceae], in Abruzzo served the same purpose. There branches were used, despite the toxicity of the plant [9,16].

***Salix alba ***L. [Salicaceae]. It has been used in tympanitis in oxen and sheep, and in myxomatosis of rabbits in Abruzzo, Latium and Marche [9,21,29,53] (see also [23]). The effectiveness is probably due to salicin as well as to chewing.

***Scrophularia canina ***L. [Scrophulariaceae]. In Latium and Abruzzo it is considered to be a cicatrizing agent for cattle etc. owing to the fact that it contains iridoid glycosides [9,29] (see also [16]).

***Sempervivum tectorum ***L. [Caprifoliaceae] in the same region [21] and ***Santolina marchi ***Arrigoni [Asteraceae] in Abruzzo [9] were also used as a digestive for cattle; the action is probably due to mucilages and resins in *Sempervivum *and to resins, essential oil, santolinon etc. in *Santolina*. The folk names of these species are respectively "rume" and "jerva de lu rume", because they are held to help rumination.

Some plants have similar uses in different European countries e.g. ***Bryonia dioica ***Jacq. [Cucurbitaceae] is considered to be a cicatrising agent both in Latium [29] and in Spain [54].

Some plants seems to be particularly agreeable as fodder:

***Urtica dioica ***L. [Urticaceae] for turkeys (a very complete and dietetic feed commonly used due to the fact that it is rich in aminoacids, proteins, minerals and vitamins and also tannins, formic and salicylic acid [52])

***Stellaria media ***L. [Caryophyllaceae], the so-called "eye of the chick" given to poultry in Latium, Marche and Tuscany. The aerial part of *Stellaria *(according to the informants) increases the laying of eggs [29,39], see also [[23] etc.], probably due to the remarkable quantity of alkaline salts, tannins, gums, a glycosidic saponine, fatty acids of the **ω**3 and **ω**6 series, antioxidant flavonoids and carotenoids [[39] and references therein].

***Convolvulus arvensis ***L. **and *Calystegia sepium ***(L.) R.Br. [Convolvulaceae], typical fodder for rabbits, stimulate the appetite [[16,39] etc.]. The latter species seems to promote better and healthier growth in rabbits [55].

***Agropyron repens ***(L.) Beauv. [Poaceae] is considered in Latium [29] and in Venetum [56] a good dietary supplement to give a shine to horses' coats. Mucilaginous compounds, triticin, and an antibiotic, agropirene [52], are present in this species.

### C) Anti-parasitic uses

These uses are due to active compounds such as essential oils (*Lamiaceae, Apiaceae*, several *Asteraceae*), sesquiterpene lactones (*Asteraceae*), alkaloids (*Solanaceae*), glycosides (*Liliaceae*), naphtoquinone compounds (*Juglandaceae*), etc. [57].

***Juglans regia ***L. [Juglandaceae]. The leaves are used as an anti-parasitic when applied to wheat in the granary. This use is widely reported for Marche, Tuscany, northern Latium, Molise, Abruzzo [29,39,58] and confirmed by dozens of informants. Wrapping a cheese with *Juglans regia *leaves repels *Tyrophagus casei *[39,47].

***Urginea maritima ***(L.) Baker. [Liliaceae]. The sliced bulb repels mice [32,41,59] and was placed in cowsheds in southern Italy to protect cattle from parasites [31].

#### Local uses

***Datura stramonium ***L. [Solanaceae]. The leaves hung in hen-houses repel parasites, a use known only in Teramo district (Abruzzo) and in Ascoli Piceno district (Marche) ([16,22]).

***Santolina etrusca ***(L.) Santi [Asteraceae], an endemic species of northern Latium and Tuscany, used only in northern Latium as an anti-parasitic in wardrobes and cupboards [39].

Convergences of anti-parasitic uses have been noted among Italian areas and countries of the Mediterranean basin, such as ***Allium cepa ***L. [Liliaceae] bulb that is rubbed onto the skin as an insect-repellent both in Spain [54] and in Latium [29]. Several anti-parasitic Italian plants are quoted for analogous uses and with often similar forms in French ethno-phytotherapy texts[37,60-64]. Other important anti-parasitic uses of plants in central Italy are summarized in a paper [47].

### D) Nutraceutics and food plants

Some food plants can play an interesting therapeutic role [29,35,37,65-67].

***Cucumis sativus ***L. [Cucurbitaceae] is used in Calabria as an expectorant and for catarrh [35].

***Helianthus tuberosus ***L. [Asteraceae], the hypoglycaemic effect is due to the inulin (Latium)[29].

***Lens culinaris ***L. [Fabaceae], is considered to be a sedative for CNS in Calabria [35].

***Phaseolus vulgaris ***L. [Fabaceae], is used as a hypotensive in Calabria [35], see also Maccioni et al. [68].

***Petroselinum sativum ***Hoffm. [Apiaceae]. In Latium, eating small quantities of the leaves promotes and restores the menstrual cycle [29].

***Plantago major ***L. [Plantaginaceae] seeds, cooked in soup as an anti-diarrhoeic in Calabria [37].

***Sonchus oleraceus ***L. and ***Sonchus ***sp.pl. [Asteraceae], eaten in several Italian regions, are cholagogue and laxative, due to their sesquiterpene lactones but also the high content of vitamin C, carotenoids and fatty acids of type **ω**-3 [69-72].

***Urtica dioica ***L. [Urticaceae], eaten after being cooked briefly this is a noteworthy dietetic food, being rich in aminoacids, proteins, mineral salts and vitamins. In Latium it is eaten as an anti-diabetic [29].

***Vicia faba ***L. [Fabaceae], the cooked seeds mashed into a purée are a remedy for colitis in northern Latium [29]. The reported effects have never been explained on the basis of their active principles and it would be interesting to carry out further studies.

The main regional uses and mixtures are summarised below, in addition to warnings regarding toxicity of some plants [65] in line with other studies [66,67].

In Latium a commonly consumed vegetable mixture, "misticanza", is prepared with young basal leaves or buds of *Tordylium apulum, Sonchus *sp.pl., *Silene vulgaris, Sanguisorba minor *and other wild species as well as the "acquacotta", a soup prepared from young shoots of boiled plants such as *Clematis vitalba, Centaurea solstitialis, Scolymus hispanicus, Nasturtium officinale *etc. [29]. Gathering young leaves and buds of wild plants for food purposes is a very ancient custom: the Latin writer Columella describes in "De Agricoltura" that at the spring equinox, various plants were collected: *Clematis vitalba, Ruscus aculeatus, Asparagus *sp. and *Tamus communis *buds and then preserved in vinegar [29]. *Urtica *sp.pl. were already used as food in ancient Greece (cited by Aristophanes) and Rome [73].

Some uses are more or less localized to single areas. The consumption of *Inula crithmoides *as food, for example, is reported only for Basilicata [74] and Campania [51]; *Asphodeline liburnica *(Scop.) Rchb. buds in omelettes are known only in Basilicata [74], while the similar *Asphodeline lutea *(L.) Rchb buds are eaten in Apulia [75] and Sicily [[76], etc.]. In southern Italy bulbs are eaten (e.g. *Ornithogalum pyrenaicum *[51,74], or the better known *Leopoldia comosa*) and tubers, such as *Asphodelus *sp.pl. [74,77], due to a past history of extreme poverty. *Scolymus hispanicus *leafy petioles are gathered in southern Italy and eaten at Easter feasts, a custom probably of Albanian origin, widespread also in southern Spain, Greece and Turkey. Other plants (e.g. *Chrysanthemum segetum*, *Reseda alba*, *Urospermum picroides *are characteristic of the area once dominated by the Greeks, and the food use is very common in Greece, according the indications given by Picchi and Pieroni [78].

Some plants could be cultivated and play a far from negligible role in developing sustainable agriculture in marginal areas: e.g. *Chenopodium bonus-henricus *L., *Taraxacum apenninum *(Ten.) Ten., *T. glaciale *Hand.-Mazz. [79].

In folk traditions many aromatic plants are often involved: e.g. *Thymus vulgaris *L. and *Satureja montana *L. [Lamiaceae] in central Italy, put in rennet, the stomach of a lamb, and added to sheep's milk give a peculiar fragrance to cheese; sometimes even being used as a substitute for curdling [21,23,29,39,80,81].

### E) Dye uses

The use of dyes based on plant pigments in central-southern Italy have long since been abandoned. In the past, they were important above all in Abruzzo (e.g. the villages of Scanno, Pescocostanzo) and in Calabria (the villages of S.Giovanni in Fiore, Longobucco). Before the importation of ***Indigofera tinctoria ***L., ***Isatis tinctoria ***L. [Brassicaceae], "pastel", furnished the blue shades given its ready availability alongside roads, in meadows and uncultivated fields and built-up areas [82]. In Scanno village warm solutions of ashes from the fireplace were used to fix the colour [16]. "Madder" is the name of ***Rubia tinctorum ***L. [Rubiaceae], that was normally used in the past; a decoction of ***Fraxinus ornus ***L. [Oleaceae] branches in Latium and Abruzzo was used to give a green colour [9,16,29]. In Latium ***Alnus glutinosa ***L. [Corylaceae] was used in dyeing hats brown [29]. In Marche region wild ***Muscari neglectum ***Guss. ex Ten. [Liliaceae], picked at the start of spring, gave a purplish tinge to hard-boiled eggs at Easter [83].

Different species of the same genus have been used in several Mediterranean countries: ***Euphorbia ***sp. div. [Euphorbiaceae] are employed to give a white colour to clothes both in Greece [84] and in Sardinia [85]; pink is furnished by ***Rubia tinctorum ***[Rubiaceae] in Greece [84] and by ***Rubia peregrina ***in Sardinia [85].

### F) Miscellaneous uses

Several home-made and handicraft uses have been quoted, e.g.:

***Ampelodesmos mauritanicus ***(Poiret) T.Dur. et Sch. [Poaceae]. Ropes, baskets etc. are made from the leaves and stems in Latium, Basilicata, Molise and Apulia regions [29,30,58,86]. In Latium the aerial parts were used by shepherds to make torches that are prepared also with *Asphodelus albus *and *Verbascum *sp.pl. [29].

***Ballota pseudo-dictamnus ***L. [Lamiaceae], in Apulia and Latium the calyx is used to support the wicks of home-made lamps [29,30,87].

***Euphorbia dendroides ***L. has been utilized in the coastal area of Basilicata in illegal fishing because of its icthyotoxic latex [32]. This use is shared also by the nearby area of the Park of Mt.Pollino [77], southern Europe [12,88] and Spain [89]. Analogous uses are described for different *Euphorbia *species in Italy, Greece and Spain.

***Ocimum basilicum ***L. leaves [Lamiaceae], together with *Rosmarinus officinalis, Prunus persica, Laurus nobilis, Prunus cerasus *leaves etc. are used in decoctions to polish and to perfume barrels of wine [9,21].

***Scrophularia auriculata *L. **[Scrophulariaceae], in southern Latium the plant was used as soap [29], while commonly the use as a detergent is reported for *Saponaria officinalis *L. (saponosides).

***Spartium junceum *L. **[Fabaceae], the use of the broom fibres (the scientific name *Spartium *from the Greek Σπαρτον = rope) to make fabrics was spread in Basilicata and Calabria [77,86] and it is documented in the Museum arbereshë (S.Paolo Albanese). This use dates back to the Phoenicians, Romans, and Greeks who used broom fibres to make sails. Today this use is still practised only near Serrastretta (CZ) in Calabria. In Latium broom branches were gathered as fuel for wood ovens [29].

***Styrax officinalis ***L. [Styracaceae], branches of this plant, widespread in Asia (e.g. Turkey) and in Italy present only near Rome, have been used to make domestic and agricultural tools [29].

***Vitis vinifera ***L. [Vitaceae], young shoots with sap are used as fuel, giving a particular fragrance to the artichokes, cooked according to a local country recipe [29].

Methods and suggestions for the study of this particular ethnobotanical field can be found in some texts [29,86], and lists of plants used for basketry and handicraft or domestic use are reported in [20,29,90].

### G) Vernacular names and toponyms

Some local plant names derive from the disease they can be used to treat.

***Ceterach officinarum ***DC. [Aspleniaceae] in Basilicata is "spaccapietre", which means "stone-breaker", and it is typically used in southern Italy for its diuretic properties to treat kidney stones (mineral salts, mucilages and tannins are present) [31,32,91].

In Abruzzo ***Anagallis arvensis ***L. [Primulaceae] is known as "jerva della fistola", (jerva = grass, fistola = sore) [22].

***Sempervivum tectorum ***L. [Crassulaceae], is indicated, as already cited as "rume"[21] in Marche (see above).

Several toponyms take their origin from plant names such as " prato (meadow) della porrara" (porro = ***Asphodelus albus ***Miller) [Liliaceae]; or "vellare" that is a vegetation typology characterized by "vella" (***Ampelodesmos mauritanicus ***(Poiret) T.Dur. et Sch.) in Marche [47]. The name of Loreto in Marche originates from Lauretum, for a wood with ***Laurus nobilis ***[Lauraceae] once present near this town. In Campania this species is the origin of the toponyms Lauro, Laurino, Laurito, Laurìa, whilst Popoli, in Abruzzo, derives its name from *Populus *trees, since this village is located along the Pescara river where many poplars grow.

Names of villages such as Carpineto, Castagneto, Cerqueto and Cerreto come respectively from *Carpinus *sp.pl., *Castanea sativa, Quercus *sp.pl. and *Quercus cerris *[20].

Local names deriving from Latin and Greek are frequent, particularly in southern Italy: in Campania "vasilicoie" is ***Ocimum basilicum ***L. [Lamiaceae], in Basilicata "nepeta " is ***Calamintha nepeta ***(L.) Savi [Lamiaceae], whilst ***Hedera helix ***L. [Araliaceae] is called "gissu" from the Greek word "kyssòs" (κισσòσ). "Milègro" is ***Fraxinus ornus ***L. from the Greek "melìa" (μελìα) [20,32,86]. In Salento (southern Apulia) ***Daphne gnidium ***L. [Thymelaeaceae] is named "piperia" from the Greek word πιπερìα [30].

In the same area of Greek domination, ***Parietaria diffusa ***Merth. et Koch and ***P. officinalis ***L. [Urticaceae] were named "hortanèmu", from χòρτον ανεμου, that is 'erba di vento' (= grass of the wind) in Calimera village, and in Soleto village it is "monòhorto", from the Greek word ανεμòχορτον (that has the same meaning) [92]. Thus we have common names that are still in current use, such as "erba ti jentu" and "erba te jentu [30,93], in Apulia and in other areas of the ancient "Magna Graecia" [94]

Some vernacular names derive also from German (Longobards), e.g. "scrascia" or "scracia" indicating the bramble (*Rubus fruticosus *L.) [25,85], and "spicanarda" for lavender (*Lavandula *sp. pl.) [9,30,95].

## Conclusion

Phyto-therapy in central and southern Italy is nowadays practised by a few elderly people who resort to medicinal plants for mild complaints, whilst toxic plants are used only externally, if at all.

The principal usual manipulations of traditional remedies are generally simple and easy to prepare: infusion, decoction, maceration, fumigations. Their efficacy can sometimes be justified not by the presence of suitable chemical compounds in the plants involved but also by the widespread practice and even by observation of the benefit drawn by the informant.

Many plants are used for identical purposes but in different ways in different investigated areas, e.g. ***Leopoldia comosa ***(L.) Parl. [Liliaceae]: in Calabria the sliced and crushed fresh bulb scales are locally applied for toothache and headache [35], whilst in Basilicata they are grated [32].

These uses, tested down through the centuries, are still considered effective today and many other similar examples can be cited. The therapeutic activities involving the same range of apparatuses almost overlap not only in regions of Italy that are far from each other, but also with uses in other nations. A clear example is given by *Marrubium vulgare *L. [Lamiaceae], whose manifold Italian uses are confirmed also in Bulgaria [96] and Tunisia (Leporatti, Ghedira a study in progress) where this plant is considered effective in cases of jaundice, gastralgia, haemorrhoids, pulmonary diseases and rheumatism. Other examples are: *Sambucus ebulus *L. and S*. nigra *L. [Caprifoliaceae], whose diuretic and anti-rheumatic properties are equally acknowledged in Bulgaria, Tunisia and Italy. *Solidago virga-aaurea *L. [Asteraceaee] is used as anti-tussive and *Rubia tinctorum *L. for gallbladder in Italy and Bulgaria.

However, other examples could be given of anti-parasitic [57] or dye uses [84,85] and even in illegal fishing [88,89] and so on.

The use of different species in different Italian areas is often justified on the basis of the local availability of plants or of the presence of typical species such as *Lupinus luteus *and *Santolina etrusca *in central Italy; *Origanum heracleoticum*, *Ceratonia siliqua *and *Citrus bergamia *in southern Italy.

With regard to the origins of the popular medicine and the other folk uses, only some uses cited by field interviews are reported in the Greek, Roman and Arabic medical texts, e.g. in a famous synthesis by Mattioli [97]. Therefore the uses gathered probably come from other medicines, e.g. from folk experimentation by the Italic people, or from medicines used by dominating or colonizing cultures.

Nowadays the therapeutic uses of plants based on certain medicinal properties prevail and, unlike the past, are far less or not at all linked with superstitious or ritual aspects. Nevertheless some ritual beliefs dating back to the ancient "Theory of the Signature" (where Signature means Symbol) are still interwoven in therapeutic practice in some strictly conservative areas (such as in northern Latium, Abruzzo and, above all, southern Italian regions such as Molise, Basilicata and Calabria), so that some plant uses do not seem to be well founded. On the contrary many other plants deserve to be taken into consideration not only from the anthropological or cultural point of view, but also as meriting further phytochemical investigation.

Ferri and Miraldi [98] in 1999 wrote: "Until about thirty years ago, ethnobotanical studies were considered minor works...Instead, it was precisely in those years that a series of data should have been registered that today have been lost to us.... In the last ten years, ethnobotany and ethnopharmacology have been the object of a renewed interest... Hardly any new pharmacologically active molecules were discovered during these research projects but the situation could be different were ethnopharmaceutical research projects to receive the same kind of funding as that enjoyed today by research into synthetic substances".

Over the thirty years our studies (as well as those of other authors) have sketched an original picture of the local ethno-biodiversity that could still be exploited by the local communities and by an intelligent form of "eco-tourism" in order to promote tenable development (e.g. typical food or herbalist products, cultivation of medicinal herbs and of typical fruit and vegetable varieties, reproduction and sale of artefacts that have fallen into disuse).

## Competing interests

The author(s) declare that they have no competing interests.

## Authors' contributions

Both the authors participated in writing the paper.

**Figure 1:**
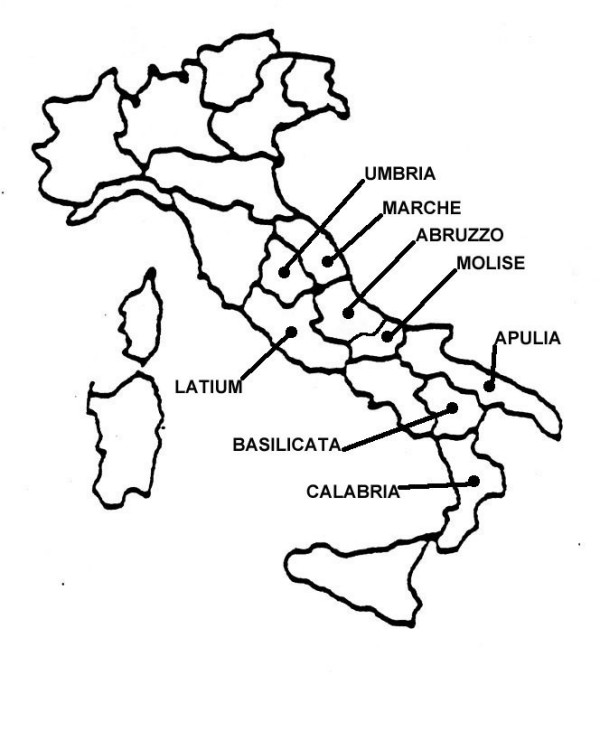
Investigated areas of Central and Southern Italy.
